# Successful AAV8 gene therapy on hepatic *ex situ* machine perfusion for mitochondrial neurogastrointestinal encephalomyopathy

**DOI:** 10.1016/j.jhep.2025.07.022

**Published:** 2025-08-06

**Authors:** Teresa Brevini, Lisa Swift, Helen Reynolds, John Ong, Richard J. Stopforth, Yogeshkumar Malam, Harry V.M. Spiers, Emilija Jovanovic, Miki Scaravaglio, Vitushan Vakeeswarasarma, James Heslop, Erin Emmett, Brock Andreatta, Emmanouil Athanasiadis, Tessa M. Cacciottolo, Claudia D. Fuchs, Michael Trauner, Sarah A. Hosgood, Teik Choon See, Pavlos Pantelis, Dora Foukaneli, William T.H. Gelson, Paul Gibbs, Kieren Allinson, Gavin J. Pettigrew, Adam Duckworth, Anna L. Paterson, Vassilis G. Gorgoulis, Richard J. Thompson, Arthur Kaser, Vasilis Kosmoliaptsis, Patrick F. Chinnery, Ramon Marti, Jelle van den Ameele, Gwilym J. Webb, Christopher J.E. Watson, Andrew J. Butler, Fotios Sampaziotis

**Affiliations:** 1 Cambridge Stem Cell Institute, Cambridge, UK; 2 Department of Surgery, University of Cambridge and NIHR Cambridge Biomedical Research Centre, Cambridge, UK; 3 Cambridge Liver Unit, Cambridge University Hospitals NHS Foundation Trust, Cambridge, UK; 4 Cambridge Institute of Therapeutic Immunology & Infectious Disease (CITIID), Department of Medicine, University of Cambridge, Cambridge, UK; 5 Department of Medicine and Surgery, University of Milano-Bicocca, Italy; 6 Synnovis Biochemical Sciences, St Thomas’ Hospital, London, UK; 7 Cambridge University Hospitals NHS Foundation Trust, Cambridge, Cambridgeshire, UK; 8 Medical Image and Signal Processing Laboratory, Department of Biomedical Engineering, University of West Attica, Athens, Greece; 9 University of Cambridge Metabolic Research Laboratories, Institute of Metabolic Science, Cambridge, UK; 10 Hans Popper Laboratory of Molecular Hepatology, Division of Gastroenterology and Hepatology, Department of Internal Medicine III, Medical University of Vienna, Vienna, Austria; 11 Department of Histology and Embryology, School of Medicine, National and Kapodistrian University of Athens, Athens, Greece; 12 Roy Calne Transplant Unit, Cambridge University Hospitals NHS Foundation Trust, Cambridge, UK; 13 Department of Histopathology, Cambridge University Hospitals NHS Foundation Trust, Cambridge, UK; 14 National Institute of Health Research (NIHR) Blood and Transplant Research Unit (BTRU) at the University of Cambridge in Collaboration with Newcastle University and in Partnership with NHS Blood and Transplant (NHSBT), Cambridge, UK; 15 Ninewells Hospital and Medical School, University of Dundee, Dundee, UK; 16 Biomedical Research Foundation, Academy of Athens, Athens, Greece; 17 Institute of Liver Studies, King’s College, London, United Kingdom; 18 Department of Clinical Neurosciences and MRC Mitochondrial Biology Unit, University of Cambridge, UK; 19 Research Group on Neuromuscular and Mitochondrial Diseases, Vall d’Hebron Research Institute, Universitat Autònoma de Barcelona, Spain; 20 Biomedical Network Research Centre on Rare Diseases (CIBERER), Instituto de Salud Carlos III, Barcelona, Catalonia, Spain; 21 Department of Medicine, University of Cambridge, Cambridge, UK; 22 Centre for Translational Stem Cell Biology, Hong Kong, China

**Keywords:** Machine perfusion, Normothermic machine perfusion, Gene therapy, Experimental hepatology, Genetic disease, Adenoassociated virus (AAV), AAV, AAV8, Liver transplantation

## Abstract

*Ex situ* normothermic machine perfusion (NMP) is rapidly emerging as a novel platform for testing therapeutics in human donor livers. Recently, perfusion of explanted patient livers was achieved, raising the possibility of using these diseased organs to increase the fidelity and resolution of drug testing and development. Here, we provide proof-of-principle for the feasibility of this approach in the context of gene therapy. We report the first successful administration of adeno-associated virus serotype 8 (AAV8) vector treatment in the explanted liver of a 34-year-old patient with mitochondrial neurogastrointestinal encephalomyopathy (MNGIE), using machine perfusion. MNGIE is caused by mutations in the thymidine phosphorylase (*TYMP*) gene, leading to nucleoside accumulation. The patient’s liver was split into anatomical left and right lobes and perfused using separate machine perfusion devices. Prior to treatment, nucleoside accumulation was observed in the perfusate of both lobes, recapitulating the cardinal feature of MNGIE. An AAV8 vector carrying the human *TYMP* gene was administered in the left lobe with the right serving as a control. AAV8 gene therapy resulted in successful vector uptake, with complete nucleoside clearance within 6 days of administration. Our results constitute the first demonstration of the efficacy of gene therapy for MNGIE in a human organ and provide proof-of-principle for using machine perfusion as a new strategy for disease modelling and testing novel therapeutics, *e.g*. gene therapy, in explanted livers.

## Introduction

A major challenge in drug development is translating preclinical findings to first-in-human studies. Up to 90% of therapeutics successfully completing preclinical testing fail in clinical trials due to a lack of efficacy and/or toxicity in patients.^1^ This discrepancy results from known limitations of existing drug screening platforms, such as the restricted capacity of *in vitro* models to recapitulate the complexity of native tissue, and inter-species variability confounding animal testing. Therefore, there is a pressing need for developing clinically relevant platforms to test high-priority candidates and bridge the gap between preclinical testing and clinical trials.

This challenge could be addressed by the rapidly emerging technology of *ex situ* normothermic machine perfusion (NMP). NMP is used in transplantation to maintain donor organs in near-physiological conditions prior to implantation, by perfusing them with warm oxygenated blood.^2^ Recently, the scope of NMP has broadened to include testing new therapeutics in animal models,^3^ evaluating donor grafts which are not used for transplantation,^4–7^ and perfusing patient explanted livers.^8^ Combining these two approaches could enable testing of novel therapeutics in explanted and perfused patient organs affected by the targeted disease, resulting in one of the most advanced and physiological systems for drug validation. Importantly, this platform could be particularly relevant in the context of rare diseases, where recruitment of adequate patient numbers to support robust clinical trials is challenging.

Here, we explore the feasibility of this strategy in the context of gene therapy for mitochondrial neuro-gastrointestinal encephalomyopathy (MNGIE). MNGIE is a rare, autosomal recessive disorder with <200 cases worldwide.^9^ It is caused by mutations in the *TYMP* gene encoding thymidine phosphorylase (TP), which is expressed in the gastrointestinal tract, nervous system, leukocytes, platelets, lungs and liver. TP metabolizes thymidine and deoxyuridine (nucleosides) to thymine and uracil, respectively. In MNGIE, TP deficiency re-sults in nucleoside accumulation causing mitochondrial DNA depletion, deletions and single nucleotide variant insertions.^9^ Mitochondrial dysfunction leads to leukoencephalopathy, myopathy, peripheral neuropathy and gastrointestinal dysmotility, resulting in malnutrition and cachexia.^9^ Symptoms are progressive, leading to death at an average age of 37 years.^9^

Treatment options for MNGIE are limited. Liver or haematopoietic stem cell transplantation have been used to restore nucleoside levels by providing a source of functional TP,^9^ which is highly expressed in these organs. However, transplantation carries mortality and complication risks, requires life-long immunosuppression, and is contra-indicated in some (*e.g*. severely malnourished) patients. Consequently, new treatments are urgently needed. Recombinant adenoassociated virus (AAV) liver-directed gene therapy, which has proven clinical efficacy for diseases such as haemophilia A and B and Crigler-Najjar syndrome,^10–12^ recently emerged as a potential new treatment for MNGIE, following successful preclinical studies in mice.^13^ Nevertheless, the efficacy of AAV gene therapy for MNGIE has never been tested in humans.

In this experimental medicine case, we use NMP to administer AAV serotype 8 (AAV8) gene therapy and correct TP deficiency in the explanted liver of a 34-year-old patient, who underwent transplantation with a liver allograft as enzyme replacement therapy for MNGIE. To characterise treatment efficacy and short-term toxicity, the liver explant was split into anatomical left and right lobe which were perfused independently for 8 days; AAV8-TYMP gene therapy was administered to the left lobe, while the right lobe was used as an internal control. Both lobes reproduced key clinical features of MNGIE during NMP, including nucleoside accumulation before treatment, while gene therapy restored physiological nucleoside metabolism in the treated lobe, resulting in thymidine and deoxyuridine clearance within 6 days.

## Materials and methods

### Ethical approval and consent

The study was performed with HRA and Health and Care Research Wales (HCRW) Approval under the Research Ethics Committee (REC) reference number 23/NS/0106. All human tissue, perfusate and blood samples were obtained under this ethical approval.

The patient was offered the opportunity to participate in the study by a consultant hepatologist in the Cambridge University Hospitals NHS Foundation Trust liver transplant waiting list clinic. After the patient expressed interest in participating, the principal investigator of the study was informed, the aim of the study was explained to the patient and informed consent was obtained 3 months prior to the start of the study. On the day of admission for liver transplantation, consent was confirmed with the patient by the principal investigator, before proceeding with the study.

### Patient information

A 34-year-old female patient with MNGIE underwent liver transplantation as metabolic replacement therapy. Details of the patient at the time of diagnosis have been reported previously.^14^ Briefly, she presented at 25 years of age with diarrhoea, vomiting, abdominal pain, and bloating. Colonic biopsies and magnetic resonance enterography were suggestive of Crohn’s disease and she was commenced on azathioprine. Treatment was complicated by headaches, myalgia, and pyrexia, leading to a brain MRI which revealed diffuse leukoencephalopathy. The combination of gastrointestinal dysmotility, cachexia, and leukoencephalopathy prompted the diagnosis of MNGIE (Fig. S1A–G). This was confirmed by the detection of elevated plasma and urine nucleosides, and by genetic testing identifying TYMP mutations (Fig. S1B–D). Reassessment of the patient’s bowel histology revealed focal atrophic changes within the muscularis mucosa consistent with MNGIE (Fig. S1F).

Other co-morbidities included eosinophilic pneumonia (controlled with prednisolone), and bile acid malabsorption. She descended from non-consanguineous parents and had three siblings; including a sister who died from MNGIE, and a living sister with MNGIE and Cowden’s disease (Fig. S1C).

Clinical deterioration with progressive ophthalmoparesis, peripheral neuropathy, allodynia, and worsening cachexia (Fig. S1G) prompted assessment for transplantation as metabolic therapy. Haematopoietic stem cell allografting was considered of greater risk than liver transplantation, therefore the patient underwent liver transplantation in the absence of significant liver disease. At the time of listing for transplantation her liver chemistry and imaging were unremarkable; BMI was only 15.5 kg/m^2^ despite nutritional supplementation (Fig. S1G).

### Hepatectomy and division of the liver

The patient received a whole liver graft from a donation after brain death paediatric donor, which was procured and transplanted following standard techniques. An inferior vena cava preserving hepatectomy was performed following sequential ligation and division of the bile duct, hepatic artery and portal vein to remove the native liver. The explanted liver was immediately flushed with cold preservation solution (Servator B University of Wisconsin Solution) and divided into anatomical left and right lobes.

### *Ex situ* liver perfusion

**Fig. 1:**
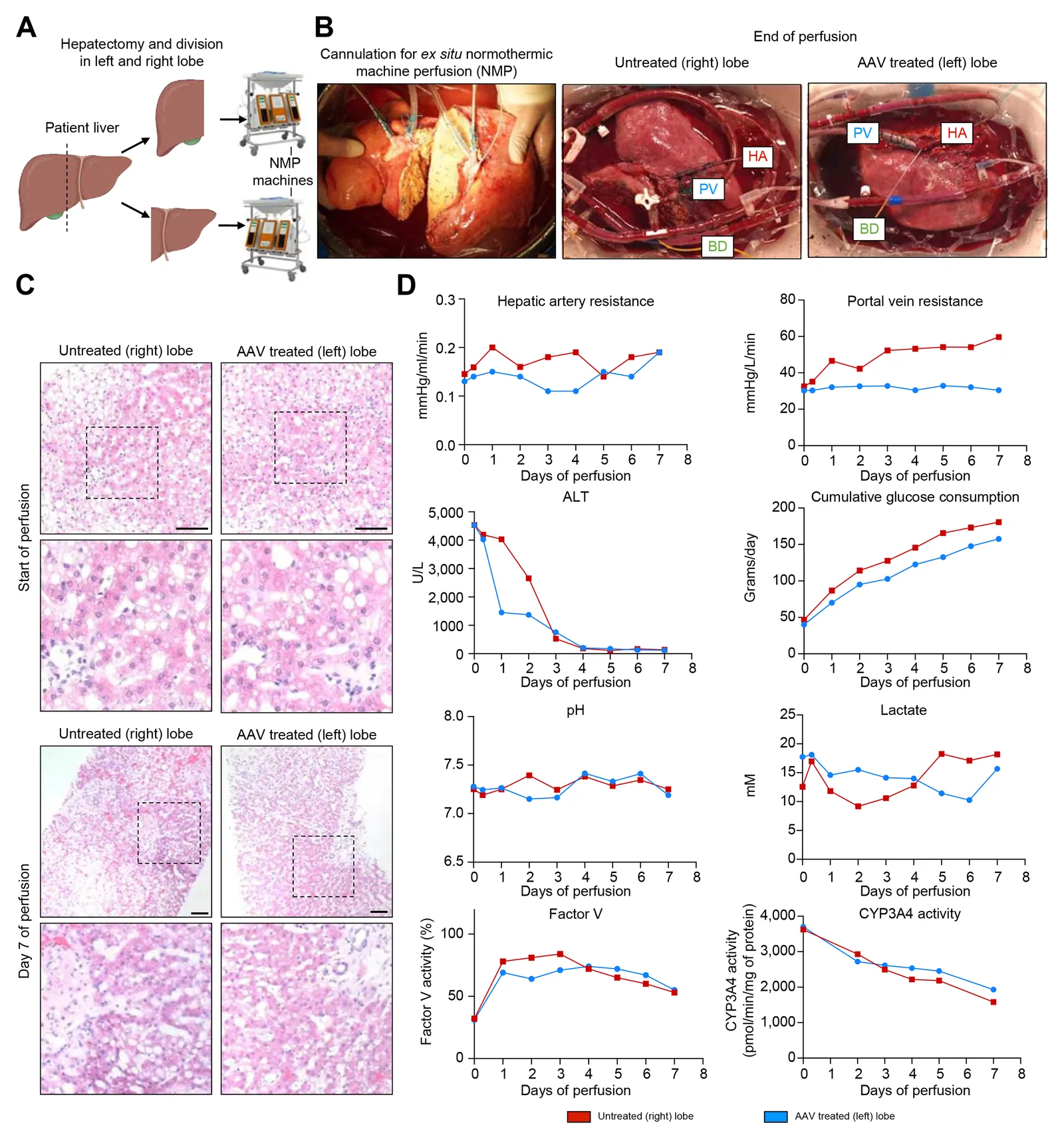
Ex situ machine perfusion of a split patient liver. (A) Schematic representation of the approach used to test AAV8 gene therapy in the patient’s explanted liver. The organ was divided into right and left anatomical lobes and each lobe was perfused on a separate ex situ machine perfusion device. AAV8 gene therapy was administered in the left lobe, while the right lobe was used as a control to benchmark the safety and efficacy of AAV8 treatment. (B) Photograph showing the left and right liver lobes following cannulation and NMP. (C) Haematoxylin and eosin staining in the two liver lobes at the beginning and end of perfusion. Scale bars 100 μm. (D) HA and PV vascular resistance, perfusate biochemistry (ALT, pH, lactate), functional parameters (cumulative glucose consumption, factor V levels, CYP3A4 enzymatic activity) showing comparable levels between the two liver lobes throughout perfusion. AAV8, adeno-associated virus serotype 8; ALT, alanine aminotransferase; BD, (hepatic) bile ducts; HA, hepatic artery; PV, portal vein. (This figure appears in color on the web.)

The portal vein and hepatic artery of each lobe were cannulated. The bile ducts were also separately cannulated and drained. The liver lobes were perfused on two separate Liver Assist (XVIVO, Groningen) perfusion devices for 7 days (Fig. 1A,B). The circuits were equipped with long-term oxygenators to sustain prolonged perfusion and equipped with a Fresenius FX 80 high flux dialyzer, connected in parallel to the main perfusion circuit, as previously described.^15^ To allow intermittent dialysis, perfusate flow through the dialyzer was controlled manually through a 3-way stopcock. Each circuit was primed with 1 L of 5% human albumin solution and three units of ABO-compatible packed red blood cells. The perfusate was supplemented with heparin (25,000 units), 8.4% sodium bicarbonate (30 ml), hydrocortisone (100 mg), calcium chloride (5 mmol), magnesium sulphate (4 mmol), Aminoven-25 (10 ml), meropenem (500 mg), fluconazole (200 mg) and continuous infusions of heparin (833 units/hour) and epoprostenol (1.7 μg/hour). To ensure adequate antimicrobial coverage throughout perfusion, meropenem (500 mg) and fluconazole (200 mg) were administered 24 and 48 hourly, respectively.

The Liver Assist utilizes a dual-pump configuration with an open venous reservoir and thermoregulatory unit. Once the two liver lobes were connected to the perfusion devices, gradual rewarming from 20 °C to 37 °C occurred over 30 minutes, according to our established clinical protocol. Perfusion parameters were set to maintain hepatic artery and portal vein pressures of 60 mmHg and 13 mmHg, respectively, and monitored throughout the experiment. Blood gas analysis was performed on the two circuits every 1-3 hours using an epoc^®^ Blood Analysis System (Siemens Healthineers AG). Based on the blood gas results, sodium bicarbonate, dextrose or calcium chloride were administered following our established clinical perfusion protocol to maintain physiological parameters (Table S1). Following nucleoside clearance, confirmed on day 6 perfusate samples, dialysis was performed for 6 hours using a Fresenius FX 80 high flux dialyzer inserted into the perfusion circuit. Intermittent rather than continuous dialysis was necessary to avoid nucleoside losses through the dialyzer, which could confound the assessment of nucleoside clearance through the liver.

### AAV8 vector administration

A commercially available AAV8 carrying the human *TYMP* gene was used (VB231031-1639dtm; VectorBuilder; Fig. S2). AAV8 was chosen as its efficacy has been demonstrated in preclinical mouse studies for MNGIE and it is one of the most commonly used AAVs for liver-targeted gene therapy. The AAV8 was diluted in 50 ml of perfusate and administered in the portal vein circulation of the left lobe as a slow bolus at a dose of 1×10^11^ vector genomes/kg of liver weight. The dose was determined based on regimens commonly employed in gene therapy clinical trials. The right lobe received a 50 ml perfusate injection as control.

### Tissue collection, histology and immunofluorescence

Human primary tissue was obtained through liver biopsies with informed consent. Samples from organ donor livers were used as controls and are referred to as healthy. Acquired tissue was processed fresh, stored at −80 °C or fixed in 10% formalin depending on the assays performed, as specified.

For histological analysis, samples were fixed in 10% formalin, dehydrated and embedded in paraffin. Formalin-fixed paraffin-embedded tissue blocks were sectioned at a thickness of 5-7 μm using a microtome and mounted on microscopy slides for further analysis. Haematoxylin and eosin staining (Sigma-Aldrich) and Sirius red staining (Sigma-Aldrich) were performed on formalin-fixed paraffin-embedded sections. All images were acquired using a ZeissApoTome2.0 with Zen Blue software (Zeiss) and interpreted by at least two blinded histopathologists.

Immunofluorescence was performed as previously described. For comparative immunofluorescence images, the tissue sections being compared were stained simultaneously, using the same antibody master mix. Immunofluorescence images were acquired using a Zeiss LSM 710 confocal microscope using ZEN 2011 SP7 software (Zeiss). The same laser power and exposure settings were used to acquire comparative images. Imagej 2.14.0/1.54f software (W. Rasband, http://imagej.nih.gov/ij) was used for image processing.

### RNA extraction and qPCR

RNA extraction was performed following manufacturer’s instructions using Sigma GenEluteTM Mammalian Total RNA Miniprep Kit (Sigma-Aldrich). qPCR data were obtained using a QuantStudio 5 384 Well Block (Thermo Fisher Scientific). At least four biological samples were used, each sample was tested in quadruplicate. Values are relative to the house-keeping genes hydroxymethylbilane synthase and glyceraldehyde-3-phosphate dehydrogenase.

### Vector copy number determination

Total DNA was extracted using the QIAGEN DNeasy Blood & Tissue kit (QIAGEN) following manufacturer’s instructions. Vector copy number was determined using a qPCR assay with primers specific for the AAV8 vector used. Vector copy number per ml of perfusate or blood was determined against a standard curve. For each tissue at least four independent biopsies were used, and each sample was tested in quadruplicate.

### Thymidine phosphorylase activity

Thymidine phosphorylase activity was measured following a previously established protocol. Briefly, liver tissue biopsies were collected, samples were homogenised, and proteins were quantified using the bicinchoninic acid method (Thermo Fisher Scientific). Next, the homogenates were incubated with 0.1 M Tris arsenate buffer (pH 6.5) and 10 mM of thymidine (Sigma-Aldrich). The reaction was terminated by adding 0.3 M NaOH and absorbance was measured at 300 nm using a SoftMax Pro 5.4.4 on a SpectraMax M2 (Molecular Devices). Enzyme activity was expressed as nanomoles (nmol) of thymine generated per hour per mg of protein. For each tissue at least four independent biopsies were used, and each sample was tested in quadruplicate.

### Nucleoside and biochemical measurements

Nucleoside and biochemical analysis were performed by clinical laboratories using standard clinical assays. Concentrations of thymidine and deoxyuridine in the patient’s blood and in the perfusate were measured using ultra high-performance liquid chromatography. Briefly, thymidine and deoxyuridine were extracted from the samples using 10% trichloroacetate which was then back-extracted using water-saturated diethylether. 2 μl of the nucleoside extract was injected onto an ethylene bridged hybrid (BEH) C18 column (1.7 μM, 2.1 × 150 mm) on a Waters Acquity ultra high-performance liquid chromatography system. Metabolites were detected by photodiode array (Waters Acquity) and identified by their retention time and characteristic absorption profile (thymidine 266 nm; deoxyuridine 260 nm).

### Statistical analysis

Results were analysed with GraphPad Prism 10 software. Data distribution was evaluated using the Shapiro-Wilk test. Depending on the normality of data distribution a two-tailed Student’s *t* test or the non-parametric Mann-Whitney test was used for comparisons between two groups, while for comparisons among multiple groups a two-way ANOVA test followed by Tukey’s test for multiple comparisons was used. Error bars represent standard deviations. For box and whisker plots: centre line, median; box, interquartile range; whiskers, range.

## Results

### Characterisation of the patient’s explanted liver

We first performed a baseline characterisation of the patient’s explanted liver immediately post hepatectomy, which could serve as a benchmark for any subsequent tests and interventions. The organ weighed 1,679 g, which was large compared to the patient’s size (height of 158.9 cm with a BMI of 15.5 kg/m^2^) and appeared steatotic. Biopsy revealed severe steatosis without definite steatohepatitis, and moderate fibrosis (Ishak score 3) in keeping with MNGIE (Fig. 1C and Fig. S3).

### Establishment of liver perfusion

The liver was then divided into anatomical right (1,032 g) and left (647 g) lobes. Each lobe was perfused independently using two separate XVIVO Liver Assist devices (Fig. 1A–B). To explore whether the two lobes were equivalent, they were assessed for 8 hours before administration of AAV gene therapy. During this period, perfusion parameters (pressure, flow), perfusate biochemistry (pH, alanine aminotransferase) and functional parameters (glucose consumption, factor V levels, CYP3A4 enzymatic activity) (Fig. 1D) were equivalent between lobes. Lactate was consistently raised in both lobes during perfusion (Fig. 1D), which is a recognised feature of MNGIE,^9^ reflecting impaired mitochondrial respiratory chain function. In view of the persistently elevated lactate levels in the patient’s serum before transplantation (5.12 ± 2.52 mmol/L), and in the absence of evidence of functional organ decline, the stable hyperlactaemia during perfusion can be attributed to MNGIE.

### NMP recapitulates clinical features of MNGIE

**Fig. 2:**
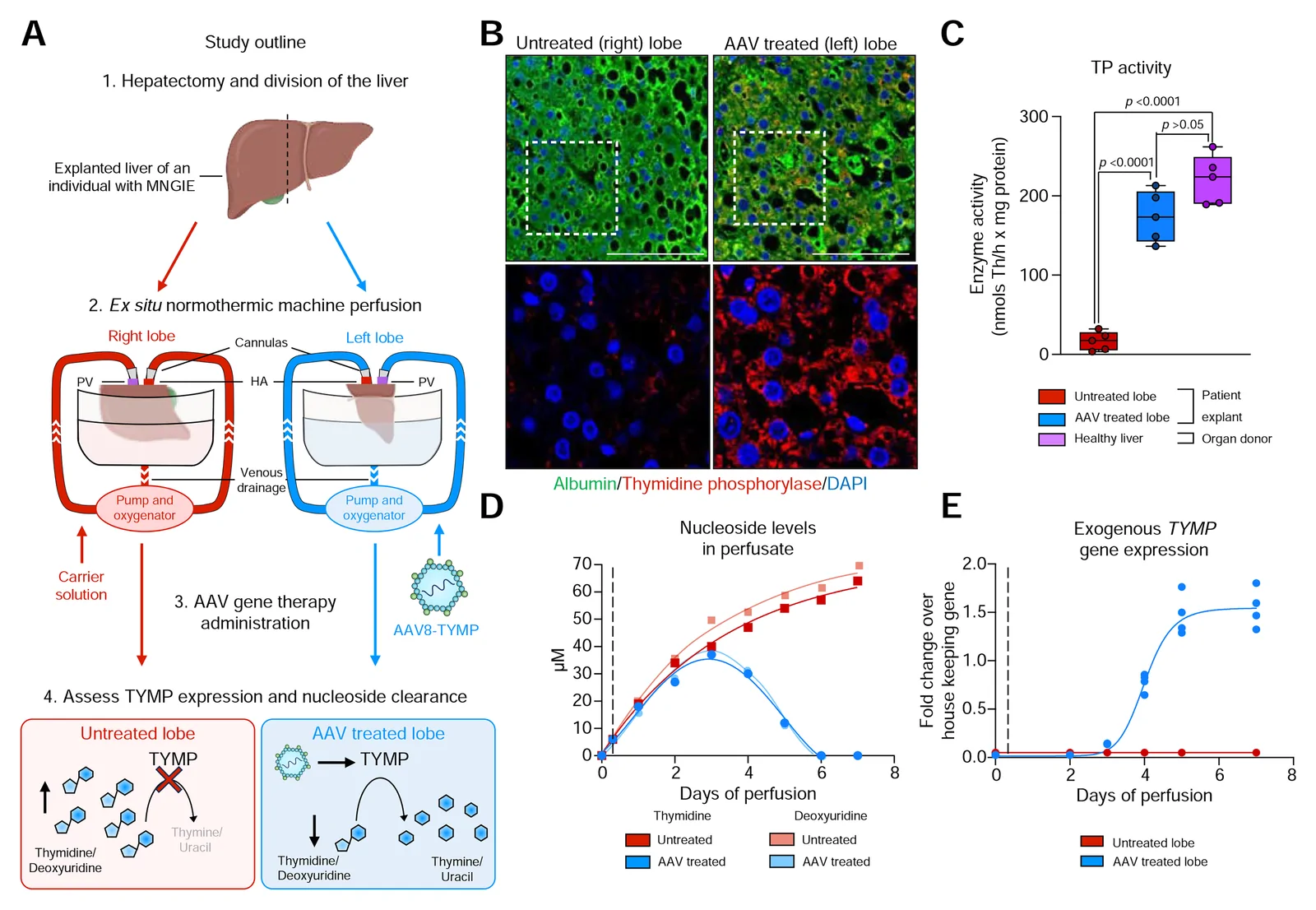
Therapeutic efficacy of AAV8 gene therapy in the explanted liver of a patient with MNGIE. (A) Schematic representation of the study performed. The native liver of a patient with MNGIE, who underwent hepatectomy for transplantation with a liver allograft, was divided into right and left anatomical lobes. Each lobe was perfused *ex situ* with perfusate for 7 days using separate machine perfusion devices. 8 hours after the start of perfusion the left lobe received AAV8 gene therapy with a vector carrying the human *TYMP* gene (AAV8-*TYMP*), while the right lobe received a perfusate injection as a control. Liver biopsies and perfusate samples for nucleoside measurements were collected at multiple timepoints throughout the experiment. (B–C) Immunofluorescence (B) and TP enzymatic activity assay (C) showing that AAV8-*TYMP* gene therapy restored TP expression and activity in the treated lobe within 3 days. Scale bars 50 µm. n = 5 liver biopsies; one-way ANOVA adjusted for multiple comparisons. (D) Nucleoside measurements in the liver lobes’ perfusate showing AAV8-*TYMP* gene therapy achieved nucleoside clearance within 6 days, reversing the disease phenotype. Day 0 corresponds to the start of perfusion; dashed lines indicate the time of AAV administration. (E) qPCR confirming expression of exogenous (vector) *TYMP* in the treated lobe, using primers spanning the TYMP-FLAG junction of the vector. n = 4 independent liver biopsy samples; one-way ANOVA adjusted for multiple comparisons. AAV8, adeno-associated virus serotype 8; MNGIE, mitochondrial neurogastrointestinal encephalomyopathy; TP, thymidine phosphorylase (protein); TYMP, thymidine phosphorylase (gene). (This figure appears in color on the web.)

Encouraged by these results, we then explored whether the patient’s perfused liver could provide an adequate disease model for testing liver-directed gene therapy by interrogating it for cardinal clinical features of MNGIE (Fig. 2A–E). Indeed, TP could not be detected on immunofluorescence, resulting in negligible TP enzymatic activity and accumulation of thymidine and deoxyuridine nucleosides in the perfusate of both lobes. These findings indicate that the explanted patient liver faithfully reproduced clinical and molecular characteristics of MNGIE during machine perfusion.

### AAV8 gene therapy corrects thymidine phosphorylase deficiency

Having established that the two perfused liver lobes recapitulated key features of MNGIE, with similar performance during NMP, we tested the efficacy of AAV gene therapy, by treating the left lobe with AAV8-TYMP and using the right lobe as a control to benchmark the rescue of TP deficiency. Vector copy number analysis in liver biopsies acquired 2 days post-virus administration revealed effective vector uptake in the treated lobe (Fig. S4). Nuclear localisation of the vector was confirmed by *in situ* hybridisation for the AAV8 DNA (Fig. S5). Vector *TYMP* expression started being detected on the second day post-treatment and steadily increased until it reached a plateau on day 5, resulting in TP protein production, which was detected via immunofluorescence within 3 days of treatment (Fig. 2B) with 18% of liver cells, predominantly hepatocytes, being positive for TP by the end of perfusion (Fig. S6). Importantly, TP enzymatic activity assays confirmed that the TP produced by the vector was functional (Fig. 2C), leading to nucleoside clearance from the perfusate of the treated lobe within 6 days of treatment (Fig. 2D).

Collectively, these results demonstrate the efficacy of AAV8-TYMP therapy for restoring nucleoside metabolism in MNGIE.

### AAV8-TYMP gene therapy is well tolerated, short-term

Both lobes were monitored and compared throughout NMP to assess the quality of the liver and interrogate the treated lobe for evidence of toxicity. Perfusion parameters (pressure, flow), perfusate biochemistry (pH, alanine aminotransferase, lactate) and functional parameters (glucose consumption, Factor V levels and CYP3A4 enzymatic activity) (Fig. 1D and Fig. S7A,B) remained equivalent between lobes. Histological analysis by a blinded histopathologist at the end of perfusion identified minor preservation changes consistent with long-term perfusion but revealed no differences between lobes or evidence of hepatitis or direct liver toxicity (Fig. 1C and Fig. S8, see supplementary information for the complete histopathology report), confirming that, during the study, AAV8 therapy was well tolerated.

## Discussion

We describe administration of AAV8 gene therapy into the explanted liver of a patient with MNGIE. Viral transduction was successful *ex situ*, restoring TP function and achieving nucleoside clearance, without evidence of toxicity. Our results support the biochemical efficacy (*i.e*. nucleoside clearance) and short-term safety of AAV8 treatment for MNGIE in a human liver and illustrate the kinetics of vector expression and nucleoside clearance after successful viral transduction. Importantly, nucleoside clearance cannot directly correct preexisting mitochondrial dysfunction. Nonetheless, animal studies and data from patients receiving liver transplantation as enzyme replacement therapy demonstrate that restoring nucleoside homeostasis can slow or halt disease progression,^9^ which illustrates the clinical value of our gene therapy approach. These data will facilitate clinical translation and inform future trials required to confirm our findings.

Our report demonstrates proof-of-principle for using *ex situ*-perfused patient organs as a platform to test new therapeutics. This is particularly relevant in rare diseases, such as MNGIE, where obtaining sufficient numbers to power robust AAV therapy trials is challenging. To overcome this barrier, we tested AAV8 treatment in the patient’s explant. Treating one liver lobe and using the other as a control enabled us to remove confounders attributed to patient-to-patient variability and obtain meaningful results with a single organ; while performing the study on the explanted organ protected the patient from risks associated with potential treatment failure and enabled almost unlimited invasive monitoring/testing.

Gene therapy for machine perfused organs could have additional clinical applications to therapeutic testing. In monogenic diseases like MNGIE, the liver may be minimally damaged beyond the genetic defect. Therefore, patient explanted livers treated with gene therapy and performing well during perfusion could be considered for use in the context of a domino transplant.

Despite these advantages, testing gene therapy in *ex situ* perfused livers also has limitations. First, it cannot interrogate the impact of treatment beyond the liver, including systemic side-effects or vector immunogenicity arising from the host immune response to the vector, which could affect treatment toxicity. Second, the pharmacokinetics and efficacy of gene therapy may differ between peripheral administration *vs*. direct delivery in the perfusion circuit. Finally, despite long perfusion, the maximum observation period of perfused livers remains relatively short (weeks) and some degree of functional decline can occur over time, highlighting the importance of carefully monitoring organ viability throughout perfusion. This could prevent adequate assessment of the long-term treatment efficacy (*e.g*. vector dilution leading to loss of transgene expression, or correction of mitochondrial dysfunction in MNGIE) and complications of gene therapy (*e.g*. insertional mutagenesis). For these reasons, *ex situ* testing of gene therapies may be better suited for bridging the gap between preclinical and first-in-human studies rather than replacing these studies. For example, data from human organs could directly inform the design of future clinical trials or complement toxicity testing experiments in non-human primates and reduce the number of animals required in accordance with the 3R principles (Refinement, Replacement and Reduction).

In conclusion, our results provide proof-of-concept for the biochemical efficacy of AAV8 gene therapy in a human liver with MNGIE and illustrate the potential applications of NMP in explanted livers as a novel disease modelling and drug screening platform. Importantly, this model fully recapitulates the complexity of human tissue while overcoming inter-species variability. This approach could be applied to a multitude of liver disorders and treatments beyond MNGIE (*e.g*. Crigler-Najjar syndrome and progressive familial intrahepatic cholestasis) and gene therapy (*e.g*. cell therapy, chemotherapeutic agents) bridging the gap between preclinical studies and clinical trials.

## Supplementary Material

Supplemental information

## Data Availability

All data are available in the manuscript or the supplementary materials.

## Highlights

- The liver explant of a patient with MNGIE was split and perfused independently for 7 days to test AAV gene therapy.
- Nucleosides accumulated in both lobes (a key feature of MNGIE) but cleared in the treated lobe following gene therapy.
- AAV gene therapy rescued TYMP deficiency in a human liver with MNGIE.
- First demonstration of the safety and efficacy of AAV8 gene therapy in a human liver using machine perfusion.
- Paradigm shift in perfusion: from donor organs to patient explants to test new treatments.

## Abbreviations

AAV(8): adeno-associated virus (serotype 8)
MNGIE: mitochondrial neurogastrointestinal encephalomyopathy
NMP: normothermic machine perfusion
TP: thymidine phosphorylase (protein)
*TYMP*: thymidine phosphorylase (gene).
