# Supplementary material for: Successful AAV8 gene therapy on hepatic ex situ machine perfusion for mitochondrial neurogastrointestinal encephalomyopathy
Source: J Hepatol. Author manuscript; Available in PMC 2026 Oct 16. (PMC7619571; doi:10.1016/j.jhep.2025.07.022)
Supplement: Supplementary file 1 [file EMS220164-supplement-Supplemental_information.pdf]

Journal of Hepatology, Volume *83*

Supplemental information

Successful AAV8 gene therapy on hepatic *ex situ* machine perfusion for mitochondrial neurogastrointestinal encephalomyopathy

Teresa Brevini, Lisa Swift, Helen Reynolds, John Ong, Richard J. Stopforth, Yogeshkumar Malam, Harry V.M. Spiers, Emilija Jovanovic, Miki Scaravaglio, Vitushan Vakeeswarasarma, James Heslop, Erin Emmett, Brock Andreatta, Emmanouil Athanasiadis, Tessa M. Cacciottolo, Claudia D. Fuchs, Michael Trauner, Sarah A. Hosgood, Teik Choon See, Pavlos Pantelis, Dora Foukaneli, William T.H. Gelson, Paul Gibbs, Kieren Allinson, Gavin J. Pettigrew, Adam Duckworth, Anna L. Paterson, Vassilis G. Gorgoulis, Richard J. Thompson, Arthur Kaser, Vasilis Kosmoliaptsis, Patrick F. Chinnery, Ramon Marti, Jelle van den Ameele, Gwilym J. Webb, Christopher J.E. Watson, Andrew J. Butler, and Fotios Sampaziotis

Successful AAV8 gene therapy on hepatic *ex situ* machine perfusion for mitochondrial neurogastrointestinal encephalomyopathy

Teresa Brevini, Lisa Swift, Helen Reynolds, John Ong, Richard J Stopforth, Yogeshkumar Malam, Harry VM Spiers, Emilija Jovanovic, Miki Scaravaglio, Vitushan Vakeeswarasarma, James Heslop, Erin Emmett, Brock Andreatta, Emmanouil Athanasiadis, Tessa M Cacciottolo, Claudia D Fuchs, Michael Trauner, Sarah A Hosgood, Teik Choon See, Pavlos Pantelis, Dora Foukaneli, William TH Gelson, Paul Gibbs, Kieren Allinson, Gavin J Pettigrew, Adam Duckworth, Anna L Paterson, Vassilis G Gorgoulis, Richard J Thompson, Arthur Kaser, Vasilis Kosmoliaptsis, Patrick F Chinnery, Ramon Marti, Jelle van den Ameele, Gwilym J Webb, Christopher JE Watson, Andrew J Butler, Fotios Sampaziotis

Table of contents

Supplementary figures……..…………………………………………………………..2

Table S1……..…………………………………………………………………..……...11

Supplementary information………………………………………………………..……12

Fig. S1. Patient clinical background.

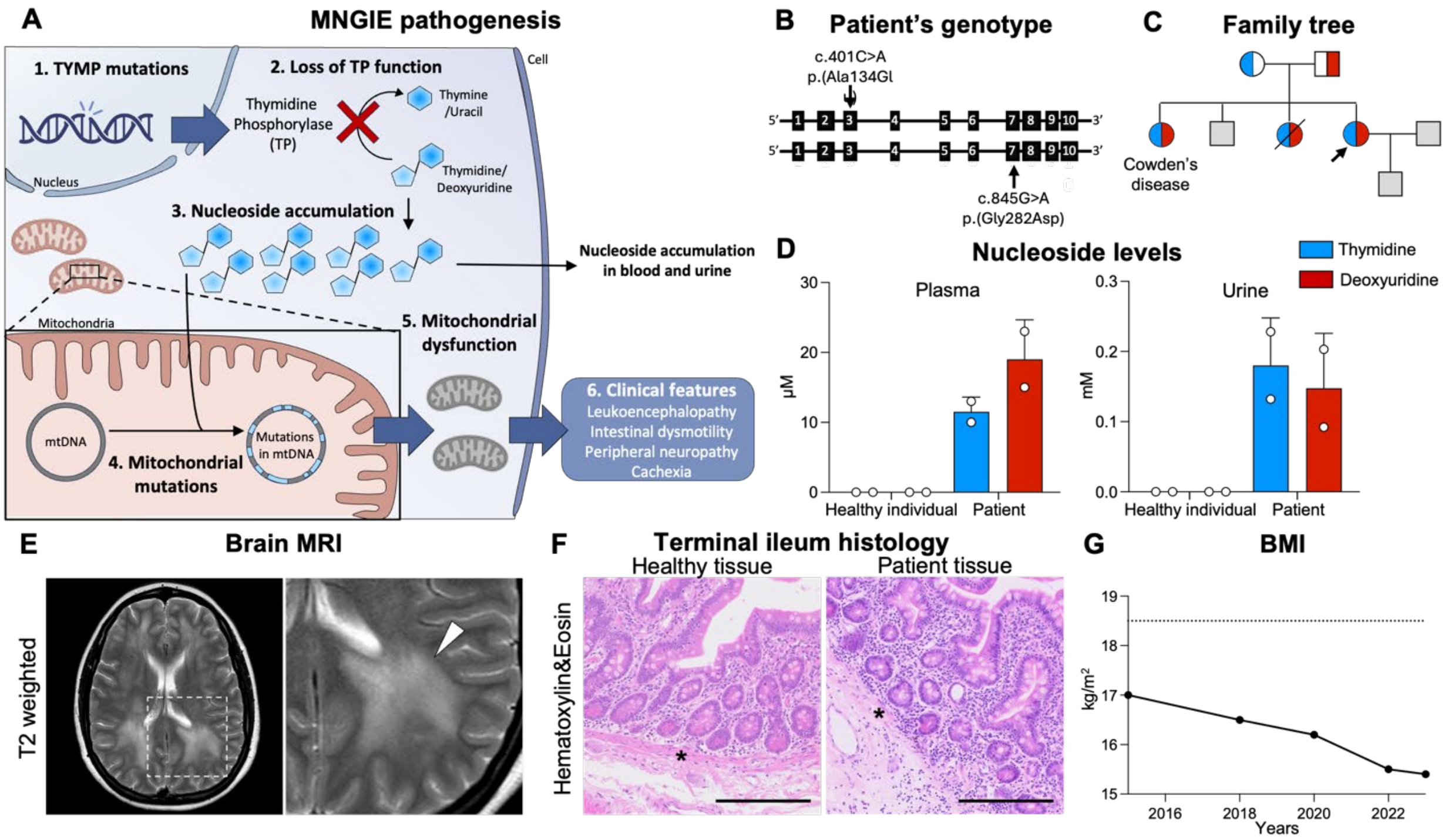

Fig. S1. Patient clinical background. (A) Schematic representation of the pathogenetic mechanism in MNGIE. *TYMP* mutations lead to lack of thymidine and deoxyuridine (nucleosides) conversion to thymine and uracil, respectively. Nucleoside accumulation results in mitochondrial DNA mutations causing mitochondrial dysfunction which leads to the clinical manifestations of MNGIE. (B) Patient's genotype identifying the patient as a compound heterozygote with one previously reported (Ala134Glu) and one novel (Gly282Asp) *TYMP* mutations. (C) Family tree showing the patient inherited the two distinct TYMP mutations from heterozygous parents. Blue represents the maternal mutation and red the paternal mutation. Grey represents untested family members. Carriers are shown with half-filled symbols. The proband is indicated with an arrow. (D) Nucleoside measurement showing accumulation of thymidine and deoxyuridine in the patient's plasma and urine as a result of TYMP mutations. N=2 independent measurements; bars, standard deviation. (E) Brain magnetic resonance imaging (MRI) showing leukoencephalopathy, with diffuse periventricular and subcortical white matter abnormality (white arrow) in the patient. (F) Hematoxylin and eosin staining showing muscularis mucosa atrophy in the patient's terminal ileum underlying gastrointestinal dysmotility. Scale bars, 100 µm. (G) Body mass index (BMI) measurement indicating

worsening cachexia and clinical deterioration, which prompted assessment for liver transplantation as enzyme replacement therapy. Healthy refers to samples from organ donors.

Fig. S2. AAV8-TYMP vector map.

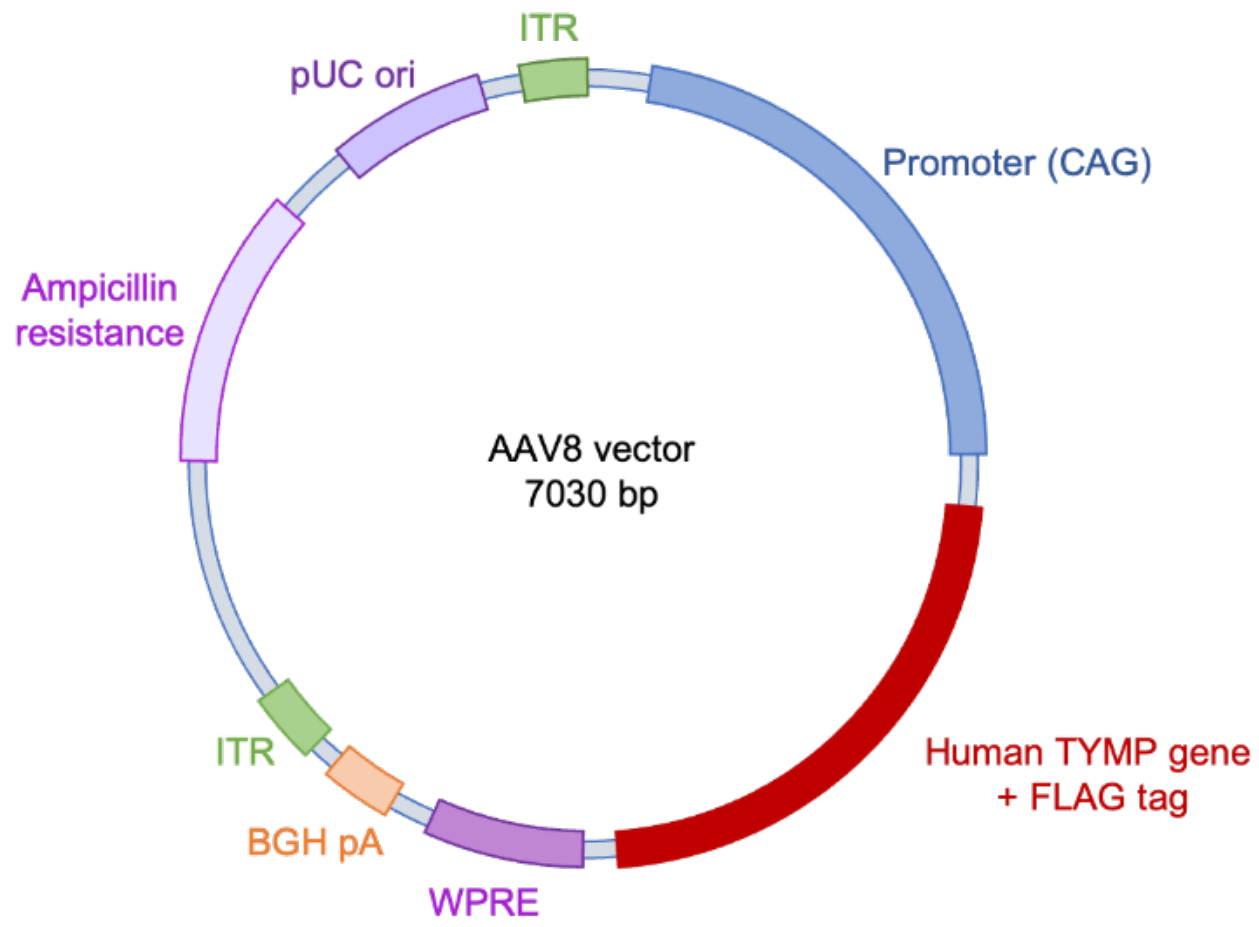

Fig. S2. AAV8-TYMP vector map. Map of the AAV8-TYMP vector employed for gene therapy.

Fig. S3. Evidence of fibrosis in the liver explant of a patient with MNGIE.

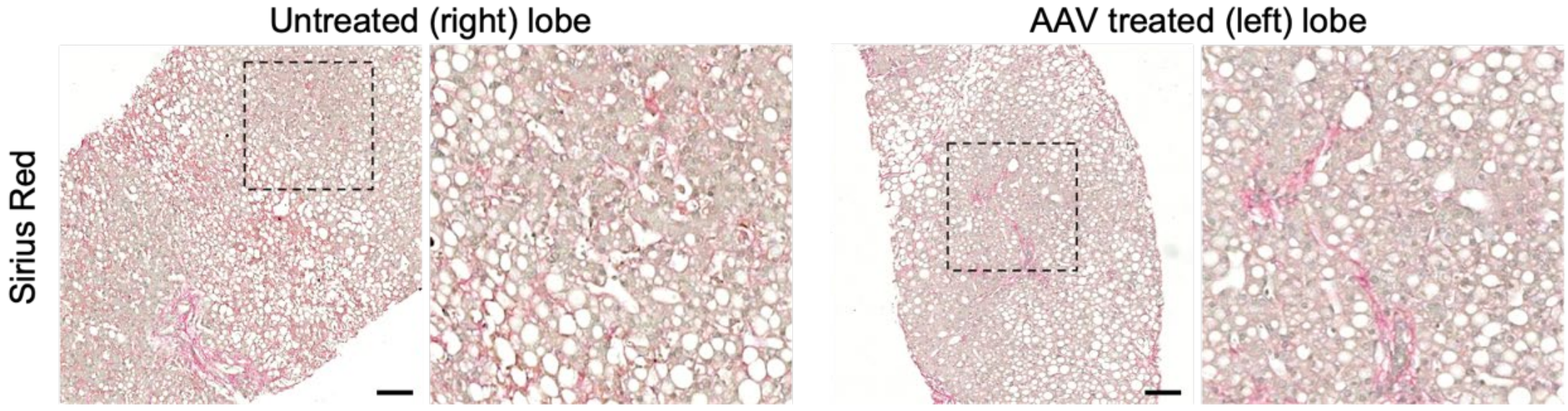

Fig. S3. Evidence of fibrosis in the liver explant of a patient with MNGIE. Sirius red staining in liver biopsies obtained at the start of perfusion showing moderate fibrosis in both lobes. Scale bars 100 μm.

Fig. S4. AAV8-TYMP vector uptake in liver biopsies.

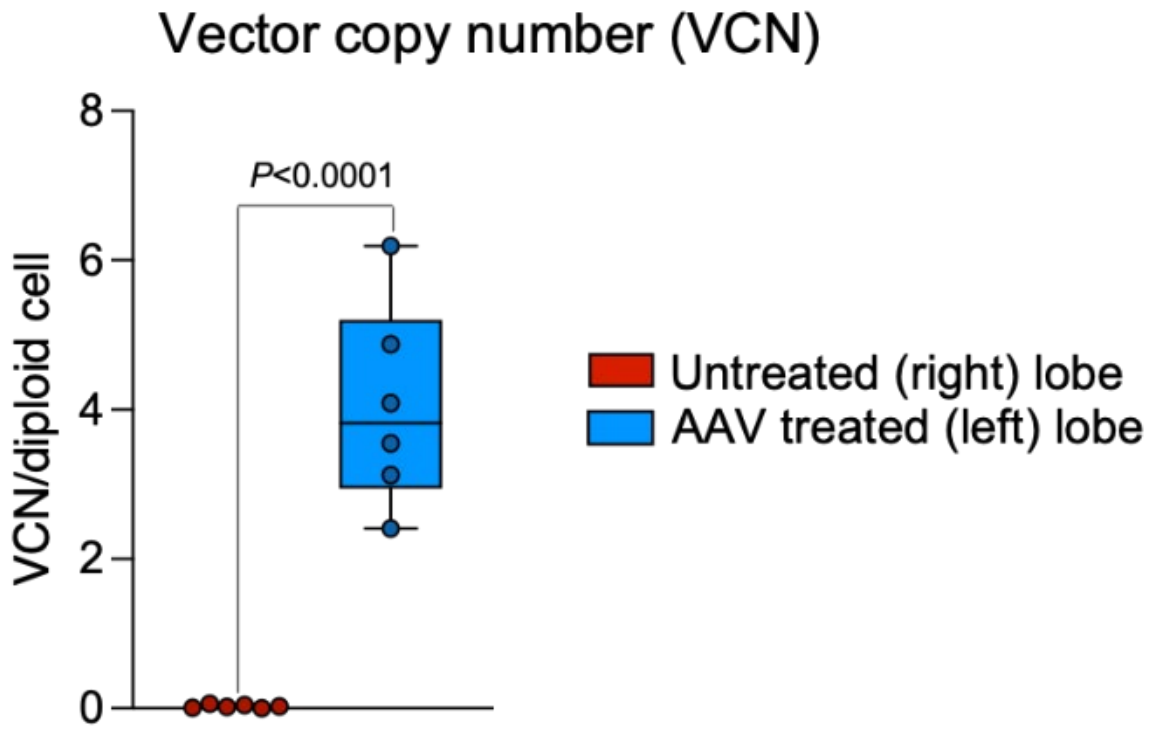

Fig. S4. AAV8-TYMP vector uptake in liver biopsies. VCN per diploid genome showing vector uptake in the treated lobe 2 days following AAV8-TYMP administration. N=6 biopsies per liver lobe; unpaired two-tailed t-test; centre line, median; box, interquartile range (IQR); whiskers, range; bars, standard deviation.

Fig. S5. AAV8 vector localisation.

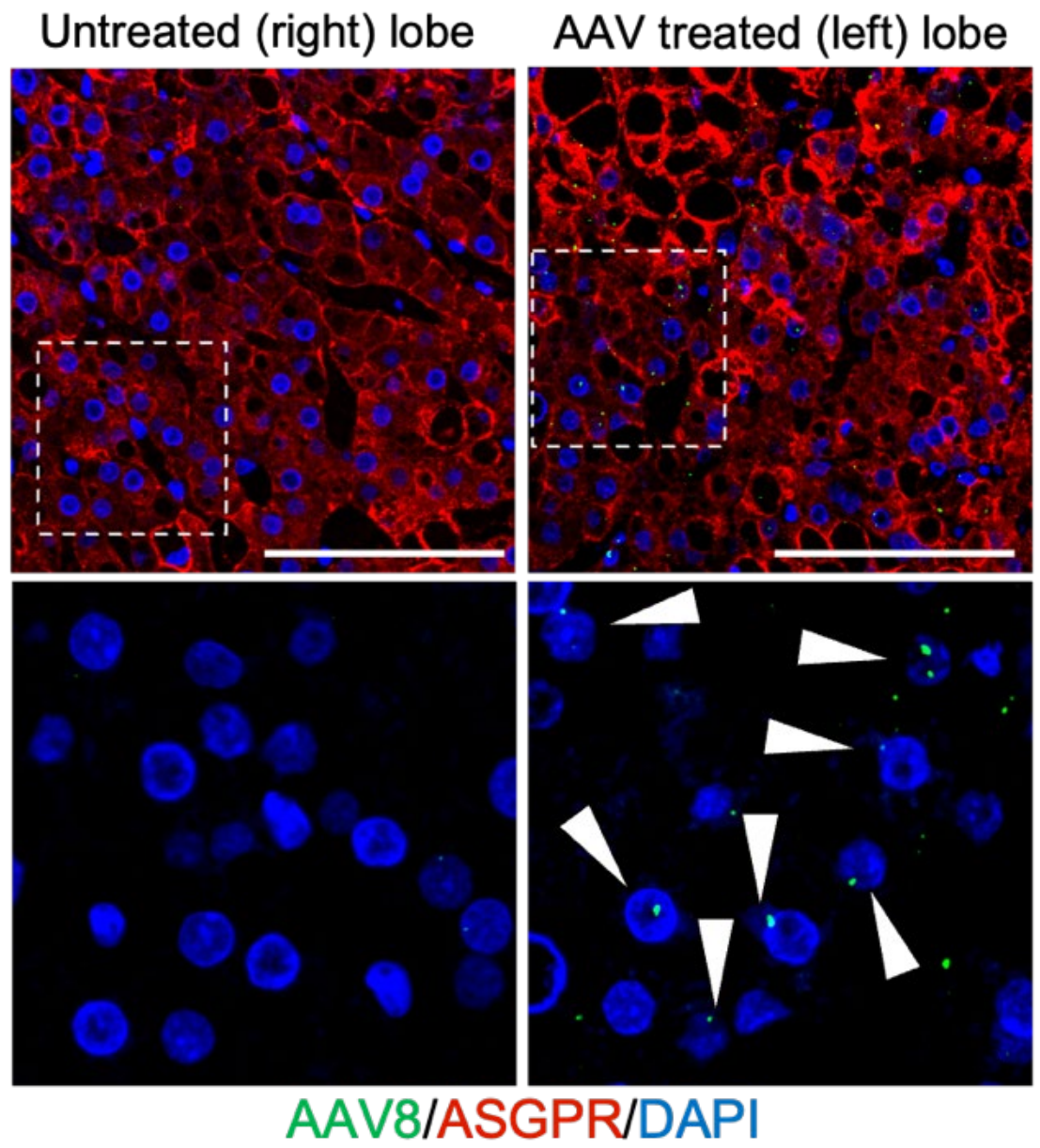

Fig. S5. AAV8 vector localisation. Representative *in situ* hybridisation (ISH) images for the AAV8-TYMP viral vector DNA in biopsy samples from each lobe of the patient's perfused liver, demonstrating predominantly nuclear vector localisation in the hepatocytes of the treated lobe. Scale bars, 100µm.

Fig. S6. AAV8 transduction efficiency.

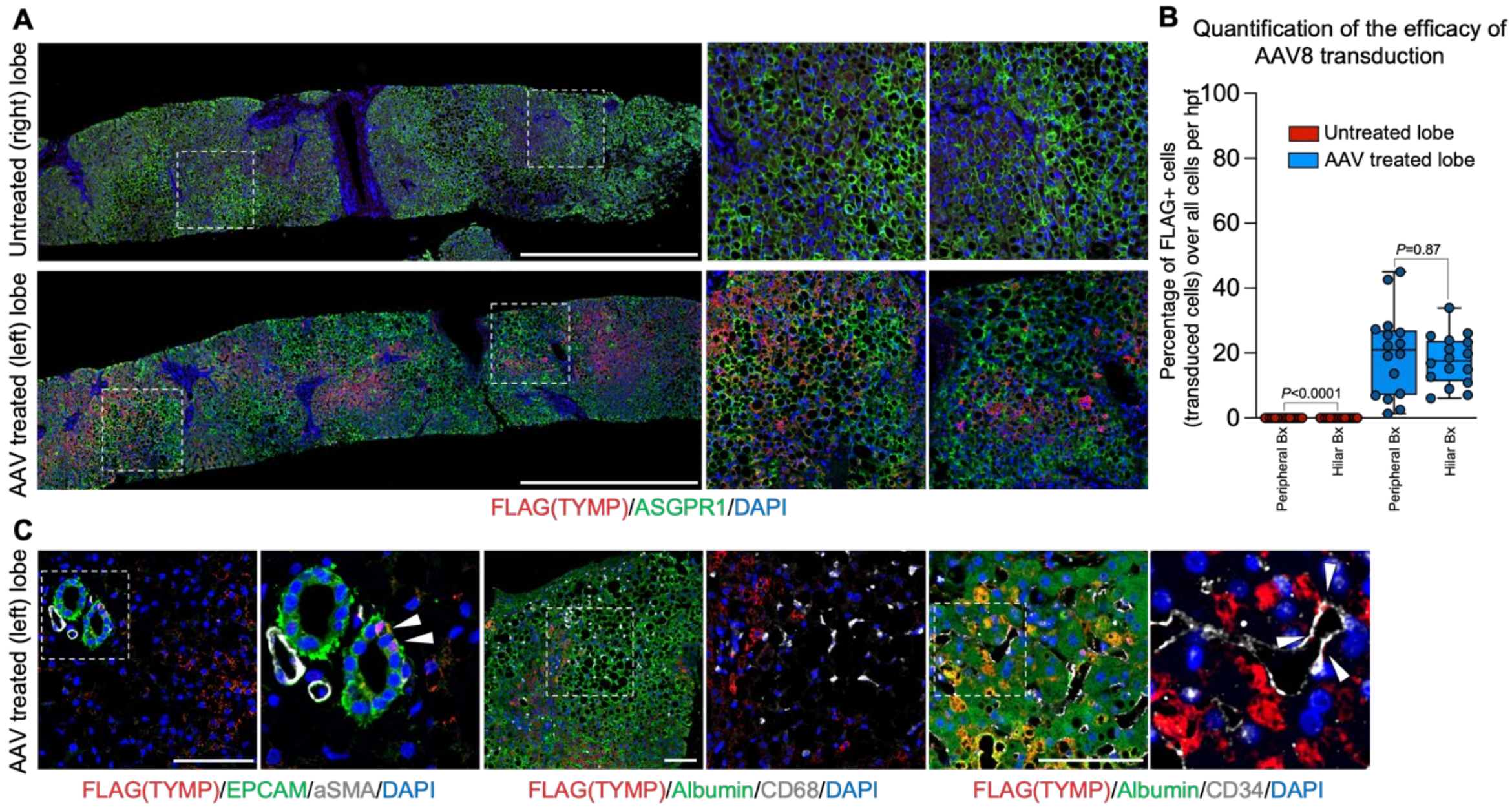

Fig. S6. AAV8 transduction efficiency. (A-B) Immunofluorescence images (A) and corresponding quantification (B) illustrating the expression of the vector TYMP, marked by a FLAG tag (FLAG(TYMP)) in biopsies (Bx) from different areas of the patient's treated liver lobe (hilar vs. peripheral). Automated quantification was performed using CellProfiler in 8 random fields per image in n=4 independent biopsies per liver lobe; one-way ANOVA adjusted for multiple comparisons. Scale bars, 1000µm. (C) IF images demonstrating the expression of the vector FLAG(TYMP) in different liver cell types. Scale bars, 100µm.

Fig. S7. Perfusate and bile biochemistry during perfusion.

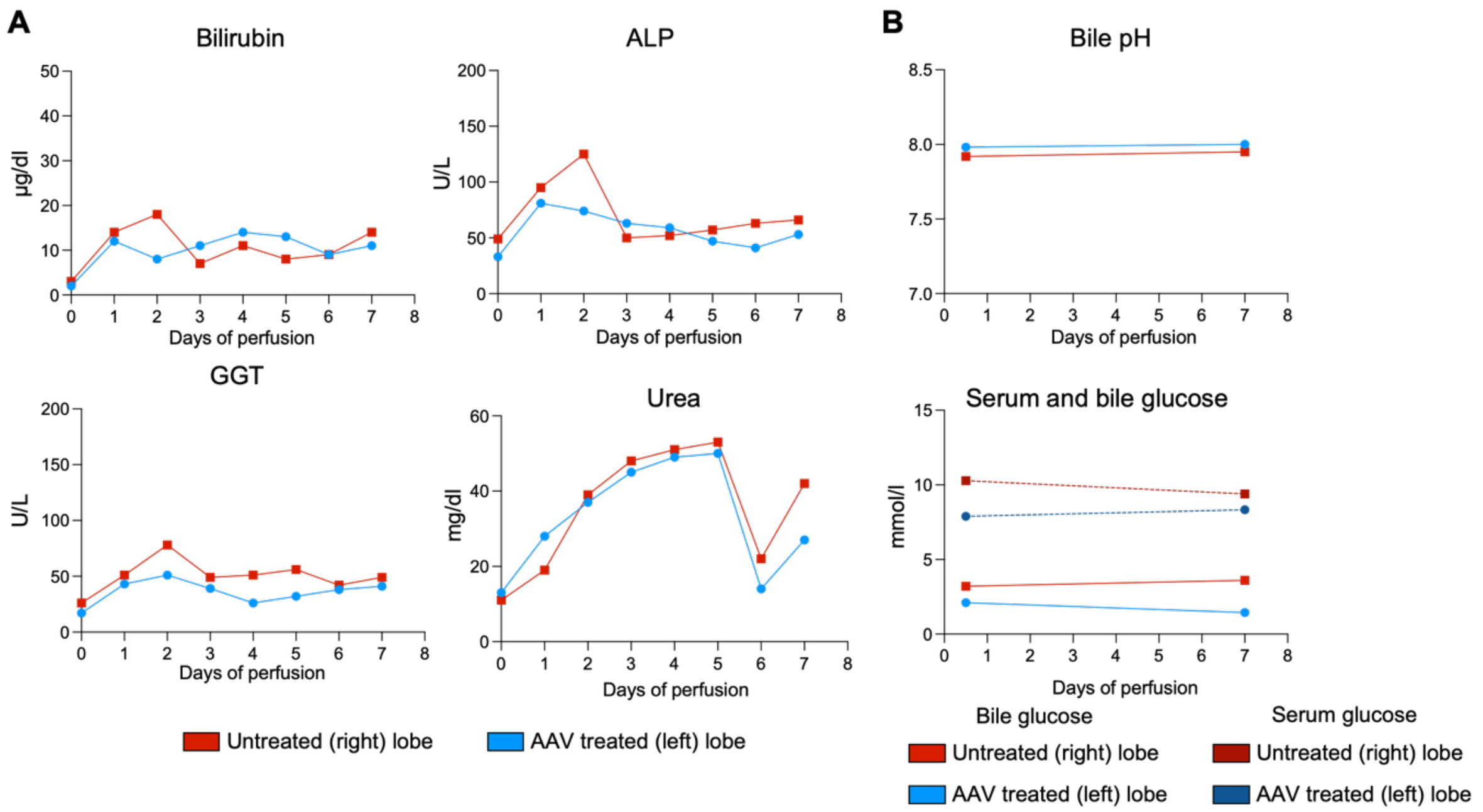

Fig. S7. Perfusate and bile biochemistry during perfusion. (A-B) Perfusate biochemistry (ALP, GGT, bilirubin, urea) (A) and bile pH and bile and serum glucose from bile samples obtained at the beginning and end of perfusion (B) demonstrating comparable values between the two liver lobes throughout perfusion.

Fig. S8. Histopathological analysis at the end of perfusion.

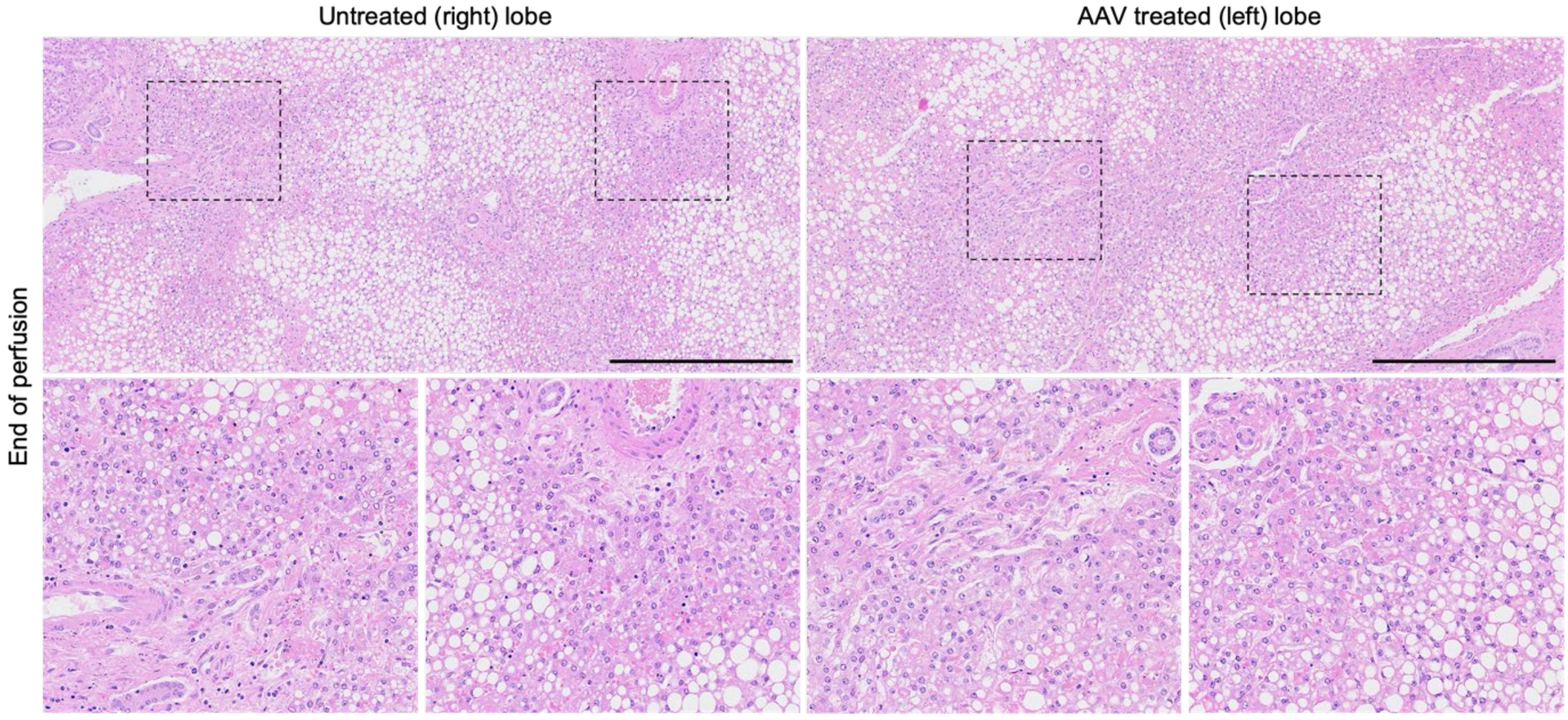

Fig. S8. Histopathological analysis at the end of perfusion. Haematoxylin and eosin images at the end of perfusion demonstrating comparable histological appearances between the two liver lobes. Scale bars 100 µm.

Table S1. Summary of infusions for long-term maintenance of livers on NMP.

Intervention	Administration
Heparin	Continuous infusion, 833 units/hour. Fresh infusion every 24 hours.
Epoprostenol	Continuous infusion, 1.7 μg/hour. Fresh infusion every 24 hours.
Meropenem	Every 24 hours, 500 mg.
Fluconazole	Every 48 hours, 200 mg.
Hydrocortisone	100 mg in prime.
Aminoven-25	Every 72 hours, 10 ml.
Magnesium sulphate 50%	2 ml in prime.
Calcium chloride	5 ml in prime.
Sodium bicarbonate	As required to ensure pH>7.3
Glucose	As required to ensure glucose levels >10 mmol/l
Dialysis	Intermittent dialysis following day 6 to avoid potential confounders in the assessment of nucleoside clearance

Supplementary information

Histopathologist report

Sections from the untreated and treated lobe overall have similar appearances. The dominant abnormality is of macrovesicular steatosis extending out from the central vein (zone 3) to involving the mid-zone (zone 2) and also in areas the periportal parenchyma (zone 1). There is no definite steatohepatitis. There are minor non-specific portal changes in the best preserved tracts. Whilst not specific this pattern of chronic liver disease is recognised to occur in association with metabolic and mitochondrial disorders including mitochondrial neurogastrointestinal encephalomyopathy (MNGIE). There is associated moderate fibrosis (Ishak stage 3). Superimposed on this are preservation changes comprising small droplet steatosis, variable hepatocyte dropout extending out from the central vein, occasional bile duct plugs, and occasional vascular microthrombi consistent with a very prolonged period of ex situ perfusion prior to fixation.
